# O.2.1-1 Development of a physical activity prescription by practitioners using a modified-Delphi method

**DOI:** 10.1093/eurpub/ckad133.106

**Published:** 2023-09-11

**Authors:** Alexis Lion, Michael J Worth, Axel Urhausen, Daniel Theisen, Charles Delagardelle

**Affiliations:** Fédération Luxembourgeoise des Associations de Sport de Santé, Luxembourg; Association Luxembourgeoise des Groupes Sportifs pour Cardiaques, L-1445 Strassen, Luxembourg; Luxembourg Institute of Research in Orthopedics, Sports Medicine and Science, L-1460 Luxembourg, Luxembourg; Fédération Luxembourgeoise des Associations de Sport de Santé, Luxembourg; Luxembourg Institute of Research in Orthopedics, Sports Medicine and Science, L-1460 Luxembourg, Luxembourg; Sports Medicine Research Laboratory, Luxembourg Institute of Health, L-1460 Luxembourg, Luxembourg; Sports Clinic, Centre Hospitalier Luxembourg - Clinique d'Eich, L-1460 Luxembourg, Luxembourg; Alan – Maladies Rares Luxembourg, L-1896 Kockelscheuer, Luxembourg; Fédération Luxembourgeoise des Associations de Sport de Santé, Luxembourg; Association Luxembourgeoise des Groupes Sportifs pour Cardiaques, L-1445 Strassen, Luxembourg; Luxembourg Institute of Research in Orthopedics, Sports Medicine and Science, L-1460 Luxembourg, Luxembourg; Department of Cardiology, Centre Hospitalier du Luxembourg, L-1210 Luxembourg, Luxembourg; alexis.lion@flass.lu

## Abstract

**Purpose:**

In Luxembourg, only a small minority of patients with chronic diseases regularly participate in therapeutical physical activity (PA) organised by dedicated associations. As recommended by the World Health Organisation (WHO), the practitioners should advise their patients to be active. However, Luxembourg practitioners only provide PA counselling to 1 in 4 of their patients. In this context, we aimed to develop a PA prescription (PAP) that would help efficiently promote PA by Luxembourg practitioners.

**Methods:**

We used a modified-Delphi method which is an iterative survey method to obtain experts opinion with the aim of establishing a consensus. In the first phase, 17 independent experts (medical doctors, sport and exercise scientists, physiotherapists) developed an initial PAP model (the front page contained the PAP, and the reverse page the WHO recommendations, examples of good practice, and contact information) and its user guide. They also created a questionnaire evaluating the front page of the PAP using 39 5-point Likert-scale questions (ranging from totally disagree to totally agree), and the reverse page of the PAP and its user guide using one 5-point Likert-scale question each. In the second phase, this questionnaire was sent to a panel of Luxembourg practitioners. The questions that had not reached consensus among the panellists were sent anew, with the results concerning these items, for re-evaluation.

**Results:**

The panel included sixty-six medical doctors. Of the 39 items on the front page of the PAP, six did not reach consensus and had to be re-evaluated in a 2^nd^ round, in which 50 practitioners participated. After this 2^nd^ round, four items still did not reach consensus and were made optional. As of the first round, a consensus was reached concerning the reverse page of the PAP and its user guide.

**Conclusion:**

This modified-Delphi study has enabled the development of a consensual and valuable tool for the promotion of PA by practitioners for practitioners. At present, this tool has yet to be implemented, preferably in the framework of a national PA promotion program that addresses the public health challenges associated with physical inactivity.

**Support/Funding Source:**

No funding.

